# SAP97 Controls the Trafficking and Resensitization of the Beta-1-Adrenergic Receptor through Its PDZ2 and I3 Domains

**DOI:** 10.1371/journal.pone.0063379

**Published:** 2013-05-16

**Authors:** Mohammed M. Nooh, Anjaparavanda P. Naren, Sung-Jin Kim, Yang K. Xiang, Suleiman W. Bahouth

**Affiliations:** 1 Department of Pharmacology, The University of Tennessee Health Sciences Center, Memphis, Tennessee, United States of America; 2 Department of Biochemistry, School of Pharmacy of Cairo University, Cairo, Egypt; 3 Department of Physiology, The University of Tennessee Health Sciences Center, Memphis, Tennessee, United States of America; 4 Department of Pharmacology, University of California Davis, Davis, California, United States of America; Hungarian Academy of Sciences, Hungary

## Abstract

Previous studies have determined that the type-1 PDZ sequence at the extreme carboxy-terminus of the ß_1_-adrenergic receptor (ß_1_-AR) binds SAP97 and AKAP79 to organize a scaffold involved in trafficking of the ß_1_-AR. In this study we focused on characterizing the domains in SAP97 that were involved in recycling and resensitization of the ß_1_-AR in HEK-293 cells. Using a SAP97 knockdown and rescue strategy, we determined that PDZ-deletion mutants of SAP97 containing PDZ2 rescued the recycling and resensitization of the ß_1_-AR. Among the three PDZs of SAP97, PDZ2 displayed the highest affinity in binding to the ß_1_-AR. Expression of isolated PDZ2, but not the other PDZs, inhibited the recycling of the ß_1_-AR by destabilizing the macromolecular complex involved in trafficking and functional resensitization of the ß_1_-AR. In addition to its PDZs, SAP97 contains other protein interacting domains, such as the I3 sequence in the SRC homology-3 (SH3) domain, which binds to AKAP79. Deletion of I3 from SAP97 (ΔI3-SAP97) did not affect the binding of SAP97 to the ß_1_-AR. However, ΔI3-SAP97 could not rescue the recycling of the ß_1_-AR because it failed to incorporate AKAP79/PKA into the SAP97-ß_1_-AR complex. Therefore, bipartite binding of SAP97 to the ß_1_-AR and to AKAP79 is necessary for SAP97-mediated effects on recycling, externalization and functional resensitization of the ß_1_-AR. These data establish a prominent role for PDZ2 and I3 domains of SAP97 in organizing the ß_1_-adrenergic receptosome involved in connecting the ß_1_-AR to trafficking and signaling networks.

## Introduction

Trafficking of G protein-coupled receptors (GPCR) is involved in signaling, desensitization and subsequent resensitization of these receptors [1]. Trafficking of GPCR is a consequence of agonist binding to the GPCR and is subdivided into several distinct processes. The major function of agonist binding to the GPCR is the activation of its specific signalosome. In the case of the ß_1_- or the ß_2_-AR, norepinephrine binding to post synaptic ß-AR activates the GTP-binding stimulatory regulatory G protein, G_s_, which in turn causes the stimulation of the effector enzyme adenylyl cyclase that catalyzes the conversion of ATP into the second intracellular messenger cyclic AMP [2].

Another effect of agonist-mediated activation of the GPCR is internalization, which is a consequence of phosphorylation of activated GPCR by G protein-coupled receptor kinases [3]. Phosphorylation of the GPCR enhances the translocation and binding of ß-arrestins, which are major nodal proteins that bind to the coat protein clathrin and along with AP2 and others promote the internalization of the GPCR [4].

Unlike the trafficking of transferrin and LDL receptors, which are constitutively internalized and recycled, GPCR undergo regulated endocytosis [1]. Agonist-mediated endocytosis of GPCR directs these receptors to endosomes in which they undergo trafficking and signaling [1], [5]–[7]. For example, signaling by internalized thyroid stimulating hormone and parathyroid hormone receptors to cyclic AMP suggests that these receptors are capable of prolonged intracellular signaling, which might be physiologically relevant [5]. After their internalization, GPCR traverse through two divergent endosomal pathways that traffic these receptors either to the membrane for another round of signaling or to late endosomes/lysosomes where GPCR are ultimately degraded [1], [6], [8].

The mechanisms that regulate whether a GPCR recycles back into the membrane or is retained intercellularly are obscure, but appear to involve specific sequences in the GPCR and a variety of GPCR interacting proteins [1], [8]. We have identified several *cis*-binding sequences in the ß_1_-AR and *trans*-binding proteins that play a major role in orchestrating the trafficking itinerary of the ß_1_-AR [9], [10]. In HEK-293 cells, the agonist-internalized ß_1_-AR recycled rapidly to the plasma membrane and this process required the type-1 PDZ sequence in the distal cytoplasmic tail of the ß_1_-AR [10].

PDZ type-1 domains are sequences that correspond to X-serine/threonine-X-Ø motifs, where X is any amino acid, while Ø is a hydrophobic amino acid [11]. They bind in a sequence specific manner to other proteins that connect these sequences to other signaling and networking molecules [8], [11]. In the human ß_1_-AR, the type 1 PDZ corresponds to Glu-Ser-Lys-Val (ESKV) and binds *in-trans* to proteins related to the membrane-associated guanylate kinase (MAGUK) protein super family, such as PSD95, SAP97, GIPC and CALs [10], [12]–[14]. We determined that binding between the ß_1_-AR and SAP97 was physiologically relevant because their interaction was required for recycling and resensitization of the ß_1_-AR [10]. In addition, the interaction between the ß_1_-AR and SAP97 was required for confining the ß_1_-AR to the plasma membrane of cardiomyocytes-like cells [15].

SAP97 and other members of the MAGUK protein super family contain numerous protein-protein interacting modules that participate in scaffolding of signal transduction networks [11], [16]. The modules of the ß-isoform of SAP97 contain an N-terminal L27 domain that can form homodimers of SAP97 or heteromultimers with other L27-containing proteins such as CASK [16], [17]. The L27 domain is followed by three PDZ-binding domains that typically bind to specific C-terminal sequences in target proteins [11], [16]. The PDZ domains are followed by SH3 and I2–I5 domains and finally by a guanylate kinase (GK) domain [16]–[19]. These domains are also protein-protein interaction domains that either bind to downstream signaling proteins, such as I3 binding to AKAP79, or bind together to form intramolecular clusters [18], [19].

In this study we have identified the ß_1_-AR binding site in SAP97 and determined that this site works cooperatively with the AKAP79-binding I3 site to promote recycling and functional resensitization of the ß_1_-AR.

## Materials and Methods

### Reagents and cDNA Constructs

The ß-isoform of rat SAP97 cDNA was mutagenized using the Quick-Change II XL kit (Agilent Technologies) to introduce unique restriction sites flanking each of the three PDZs of SAP97. These restriction enzymes were selected because they were not found in SAP97 and generate blunt-ended cDNAs that can be fused together to create the desired SAP97 PDZ deletion construct. HpaI and PmlI sites were introduced at positions 660 and 924 flanking PDZ1. An AfeI site was introduced at position 1200, which lies downstream of PDZ2. A BsrB1 and SnaB1 sites were introduced at positions 1380 and 1644 to flank PDZ3. The expected lengths of PDZ1-PDZ3 were 88, 92 and 88 amino acids, respectively. Finally, KpnI and ApaI sites were introduced at the 5′- and 3′-ends of SAP97 by PCR. This modified SAP97 construct was used to create the various PDZ deletion mutants. Individual SAP97 PDZs fused to GST were generated by PCR and cloned pGEX-4T-2. Cloning the SmaI-XhoI fragment of the human ß1-AR into pGEX-4T-2 generated a N-terminal GST fusion to the carboxy-tail of the human ß1-AR encoding amino acids 425–477. In addition the carboxy-tail of the ß_1_-AR was cloned into the maltose binding protein (MBP) pMAL p5X vector (New England Biolabs, Ipswich, MA). Cy3-conjugated to anti FLAG M2 IgG was purchased from Sigma (St. Louis MO). Anti-SAP97 antibody from Stressgen (Enzo Life Sciences, Farmingdale, NY), anti Pan PDZ antibody (MABN72, Millipore, Billerica, MA) anti-ß_1_-AR antibodies, for human (sc-567) or rodents (sc-568), were purchase from Santa Cruz Biotechnology (Santa Cruz, CA), AKAP79 from BD, (Franklin Lakes, NJ). The siRNA sequence to human SAP97, 5′-GATATCCAGGAACATAAAT-3′ or its control 5′-CCATAATACAAGGTATAA-3′ were cloned into two vectors, one was the pcDNA6.2-GW-miR vector and the other was the pcDNA6.2-GW/EmGFP-miR vector to generate shRNA and EGFP-shRNA constructs, respectively (Invitrogen, Grand Island, NY).

### Acid Strip Confocal Recycling Microscopy Protocol

HEK-293 cells stably expressing the FLAG-tagged WT ß_1_-AR with hSAP97 shRNA were incubated with Cy3-conjugated, anti-FLAG M2 IgG (4 µg/ml) for 1 h at 37°C. Cells were treated with 10 µM isoproterenol for 30 min at 37°C to promote agonist-mediated receptor internalization. Then the cells were chilled to stop endocytosis and exposed to 0.5 M NaCl, 0.2 M acetic acid (pH 3.5) for 4 min on ice to remove antibody bound to extracellular ß_1_-AR [10], [20], [21]. Cultures were then incubated with culture medium supplemented with 100 µM of the ß-antagonist alprenolol at 37°C for 15, 30 or 60 min to establish the recycling time. After each time period, the cover slips were rinsed and fixed in 4% paraformaldehyde with 4% sucrose in PBS (pH 7.4) for 10 min at room temperature. At the completion of recycling (i.e. the 60 min slide) the slide was exposed to a second acid wash to strip the Cy3-labeled antibody from the externalized receptor population and then fixed [20]–[22]. Confocal fluorescence microscopy was performed on coded slides using a Zeiss Axiovert LSM 510 (100×1.4 DIC oil immersion objective) and the immunocytochemical data were analyzed to determine the recycling time [9], [10], [22], [23]. To calculate the recycling kinetics, a boundary was drawn around the inner circumference of cells in order to determine the distribution of pixels between membranous and intracellular compartments. The density of the pixels residing inside the boundary versus those residing outside the boundary was used as an index for internalized and membranous ß_1_-AR, respectively. Pixels were plotted as a function of time after the removal of isoproterenol in order to calculate the recycling kinetics of the ß_1_-AR [9], [22].

### Co-Immunoprecipitations and Pull-down Assays

Cells expressing the appropriate constructs were lysed in radioimmune precipitation (RIPA) buffer (150 mM NaCl, 50 mM Tris, pH 8.0, 5 mM EDTA, 1% triton X-100, 10 µg/ml leupeptin, 10 µg/ml aprotinin, 10 µg/ml chymostatin and 1 mM phenyl methyl sulfonyl fluoride). Equal amounts of clarified lysates were added to ∼5 µl M2 anti-FLAG-agarose beads. Control experiments were performed by incubating lysates with pre-immune IgG at the same concentration for 4 h at 4°C. The immune complexes were washed three times in RIPA buffer, eluted and the eluates were subjected to Western blotting.

Rat hippocampal crude membrane fractions were prepared in the presence of protease inhibitors and washed by resuspension and re-centrifugation in RIPA buffer without detergents. Washed membranes were solubilized in complete RIPA buffer and the cleared lysates were used for immunoprecipitation as described above. Neonatal cardiac myocytes were isolated from newborn wild type FVB mouse pups and cultured in 60-mm dishes for 24 h [24]. Myocytes were infected with adenovirus (moi 100) to express the HA-tagged ß_1_-AR. After 48 hrs, the myocytes were lysed in RIPA buffer with protease and phosphatase inhibitors. The clarified lysate was mixed with 12 µl of anti-HA affinity beads for 24 h at 4°C. The resin was eluted and equal amounts of eluate were subjected to Western blotting and probed with anti SAP97, anti ß_1_-AR antibody and anti-AKAP150 antibody.

For pull-down assays, GST-fusion proteins were purified from BL21 bacteria by affinity chromatography on glutathione- or amylose-conjugated resins and then dialyzed in PBS containing 10% glycerol. HEK-293 cells expressing the various SAP97 constructs were lysed with 0.2% Triton X-100 in PBS supplemented with protease inhibitors and then clarified by centrifugation at 16,000×g*_av_* for 30 min at 4°C. GST or GST-ß_1_-AR_(425–477)_ or MBP-fusion proteins were incubated with cell lysates along with 10 µl-of glutathione- or amylose-agarose beads for 2 h at 4°C. After washing three times with lysis buffer, the proteins were eluted from the beads with 2X-laemmli sample buffer containing 20 mM dithiothreitol. Eluates were separated on SDS polyacrylamide gels, electroblotted to nitrocellulose filters and analyzed for SAP97 by immunoblotting.

Far-western assays were performed by spotting 10 µl or 20 µl of a 1 µg/ml solution of GST or GST-PDZ onto nitrocellulose filters. The filters were blocked for 1 h with 3% bovine serum albumin in TBST and then incubated with 16 µg/ml or ∼0.34 µM of purified MBP-ß_1_-AR_(425–477)_ fusion in hybridization buffer (2% bovine serum albumin in TBST) for 14 h at 4°C [23]. After washing, bound ß_1_-AR_(425–477)_ was detected by the anti-ß_1_-AR antibody.

### Biotinylation Assay of ß_1_-AR Recycling with Cleavable Biotin

Cells expressing the WT ß_1_-AR were transfected with PDZ2 of SAP97 in pIRES-EGFP or with K323A/326A-PDZ2 in pIRES. The cells were surface-biotinylated with 1.5 mg/ml sulfo-NHS-SS-biotin (Pierce) in Hanks’ balanced salt solution with Ca^2+^ and Mg^2+^ at 4°C. Biotinylated cells were exposed to isoproterenol for 30 min and then cooled to 4°C to stop membrane trafficking, and the remaining surface biotin was quantitatively cleaved with glutathione. After cleavage, warm DMEM was added, and cells were incubated at 37°C for 15, 30, and 60 min to allow internalized receptor to recycle before the cells were cooled to 4°C and incubated with glutathione cleavage buffer for a second time to ensure complete cleavage of any newly appearing surface biotin. At the end of each time point, the cells were lysed and processed as described [10], [23].

### Adenylyl Cyclase Assays for ß-AR Desensitization and Resensitization

HEK 293 cells stably expressing the WT ß_1_-AR and either the scrambled shRNA-green fluorescent protein (GFP) or hSAP97 shRNA-GFP were used to determine the effect of SAP97 on desensitization and resensitization of adenylyl cyclase activities. In addition, in cells stably expressing the WT ß_1_-AR, we determined the effect of transiently transfected PDZ2-in pIRES-EGFP or K323A/326A-PDZ2 in pIRES on desensitization and resensitization. In all these experiments, the cells were cultured on 15 cm plates that were divided into four sets. The first and second sets were used as control for desensitization and the third and fourth sets for resensitization assays. For desensitization, cells were exposed to 1 mM ascorbic acid (control) or 10 µM isoproterenol for 30 min at 37°C, and then processed for the preparation of membranes as described [9], [23]. The third set was used as the control for resensitization and the fourth set for resensitization assays. Cells for resensitization were exposed either to 1 mM ascorbic acid (control) or to 10 µM isoproterenol for 30 min at 37°C and then incubated with 100 µM alprenolol for 1.5 h at 37°C, followed by the preparation of membranes. Adenylyl cyclase activities in freshly prepared membranes were determined [22], [23] and the percentile of the K_act_ ± S.E. for isoproterenol versus 5 µM forskolin was calculated.

### Statistics

All data are expressed as means ± SE, except where indicated. For comparison between two groups of data, Student’s unpaired *t*-test was used to determine significance, while multiple groups were compared by one-way (ANOVA) with Newman-Keuls post-hoc tests using Prism software (GraphPad, San Diego).

### Computational Modeling

X-ray crystal structure of SAP9797 PDZ2 bound to the GluR-A_18_ peptide (PDB ID: 2G2L) was used as a template for computational modeling [25]. Model of the C-terminal ESKV sequence of the human ß_1_-AR was built based on the backbone coordinates of the GluR-A_15-18_ using AMBER89 force field in built-in homology modeling suit of MOE software (MOE 2011.10, Chemical Computing Group). The resulting peptide was checked by MOE’s Protein Geometry Stereo-chemical Quality Evaluation tools in order to confirm that the model’s structure is consistent with crystallographic data on similar sequences. ESKV was then substituted for GluR-A_15–18_ in template PDZ-peptide complex [25]. The energy of the complex was minimized to the Root Mean Square Gradient of 0.05. The presence of hydrogen bonds was predicted by MOE built-in function. No bonding was considered if predicted bonding energy was below 0.3 kcal/mol.

## Results

### Identification of the PDZ2 Binding Domain in SAP97 as a Binding Motif for the Carboxy-tail of the ß_1_-AR

**Figure 1:**
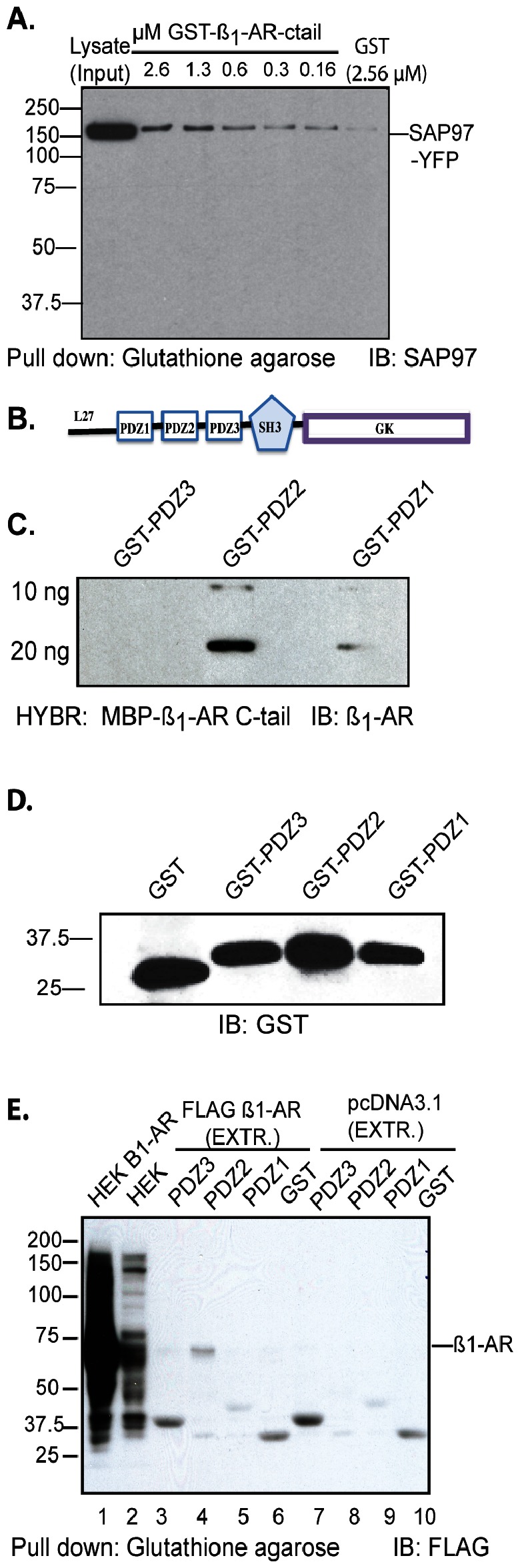
Identification of PDZ2 of SAP97 as a binding partner to the C-tail of the ß_1_-AR. *A*, increasing concentrations of GST-tagged ß_1_-AR_C-tail_ or GST were screened for interactions with SAP97 by GST pulldown using lysates from KEK-293 cell expressing SAP97-YFP. GST pull-downs (IP) were immunoblotted (IB) for SAP97 using a SAP97 antibody from Enzo Life Sciences. The optical density of each band was plotted against µM of ß_1_-AR protein to estimate the concentration of ß_1_-AR protein at 50% of the maximal optical density. These values from *n* = 3 experiments were used to calculate the apparent EC_50_ of their interaction. Input represents ∼4% of the lysate. *B*, schematic diagram of the protein-protein interacting domains of SAP97. *C*, 10 µl or 20 µl of a 1 µg/ml stock solution of GST-tagged PDZ1, PDZ2 or PDZ3 of SAP97 were slot-blotted onto nitrocellulose membranes. The membranes were hybridized with 16 µg/ml (0.34 µM) of MBP-ß_1_-AR_(425–477)_ fusion protein. The binding of the ß_1_-AR to each PDZ was detected with the sc-567 anti-ß_1_-AR antibody (Santa Cruz). *D*, Western blot (IB) of purified GST or GST-PDZ1, -PDZ2 or -PDZ3 fusions. *E,* Equal amounts of protein lysates from HEK-293 cells expressing either the empty pcDNA 3.1 or FLAG WT ß_1_-AR were mixed with 10 µg of GST or 12.5 µg of the indicated GST-tagged PDZ in a total volume of 1 ml (0.38 µM of GST or GST-PDZ). Then ∼4% input lysates (*lane*s 1, 2) or GST pull-downs (IP) from cells that expressed the WT ß_1_-AR (*lanes* 3–6) or the empty vector (*lanes* 7–10) were subjected to Western blotting (IB) and probed with the anti-FLAG antibody.

In Gardner *et al* [10] we reported that SAP97 binds to the type 1 PDZ ESKV in the carboxy-terminus of the ß_1_-AR, but the affinity of this interaction was not determined. Therefore, we mixed increasing concentrations of purified GST-ß_1_-AR_(425–477)_ or GST with lysates prepared from HEK-293 cells over-expressing SAP97-YFP (Fig. 1A). Glutathione-Sepharose pull-downs were subjected to Western blotting to estimate the relative optical densities of pulled-down SAP97-YFP at each concentration of GST-ß_1_-AR C-tail. Half-maximal optical densities from *n = 3* experiments were used to calculate the EC_50_ for the binding. These experiments indicated that SAP97 bound the carboxy-tail of the ß_1_-AR with an apparent EC_50_ = 0.5±0.2 µM.

The domain(s) in SAP97 that bind to the type-1-PDZ of the ß_1_-AR have not been determined, but are thought to involve one or more of the three PDZ domains of SAP97 (Fig. 1B). To identify the ß_1_-AR binding domain, increasing amounts of GST or GST-fusions of PDZ1-, PDZ2- or PDZ3 of SAP97 were slot-blotted to nitrocellulose membranes and hybridized with 16 µg/ml (0.34 µM) of MBP-ß_1_-AR c-tail fusions (Fig. 1C). These experiments indicated that 10 ng of PDZ2 was sufficient to bind to the c-tail of the ß_1_-AR and that maximal binding was attained by 20 ng of PDZ2. The carboxy-tail of the ß_1_-AR did not bind to PDZ3 and its binding to PDZ1 was rather low, indicating that the binding we observed in Fig. 1A was mostly due to PDZ2.

Next we expressed and purified the individual GST-SAP97 PDZs and demonstrated that these proteins migrated with expected M_r_ values (Fig. 1D). Then equal amounts of protein lysate prepared from HEK-293 cells overexpressing either the empty pcDNA 3.1 vector or the full length WT FLAG ß_1_-AR were mixed with 10 µg of GST or 12.5 µg of GST-fusions of PDZ1-, PDZ2- or PDZ3 in a total volume of 1 ml (Fig. 1E). GST pull-down followed by Western blotting with anti-FLAG IgG showed that PDZ2 of SAP97 was associated with the 65-kDa ß_1_-AR (Fig 1E, *lane* 4). These FLAG immunoblots also revealed that the 65-kDa FLAG-ß_1_-AR was the major immunoreactive band in the input from ß_1_-AR expressing HEK-293 cells (Fig. 1E, *lane* 1). However, in order to detect specific PDZ-ß_1_-AR interactions in *lanes* 3–10, the input from HEK-293 cells in *lane* 2 was overexposed revealing some non-specific immunoreactive proteins.

### Effect of SAP97 Knock down and its Rescue on Trafficking and Resensitization of the ß_1_-AR

**Figure 2:**
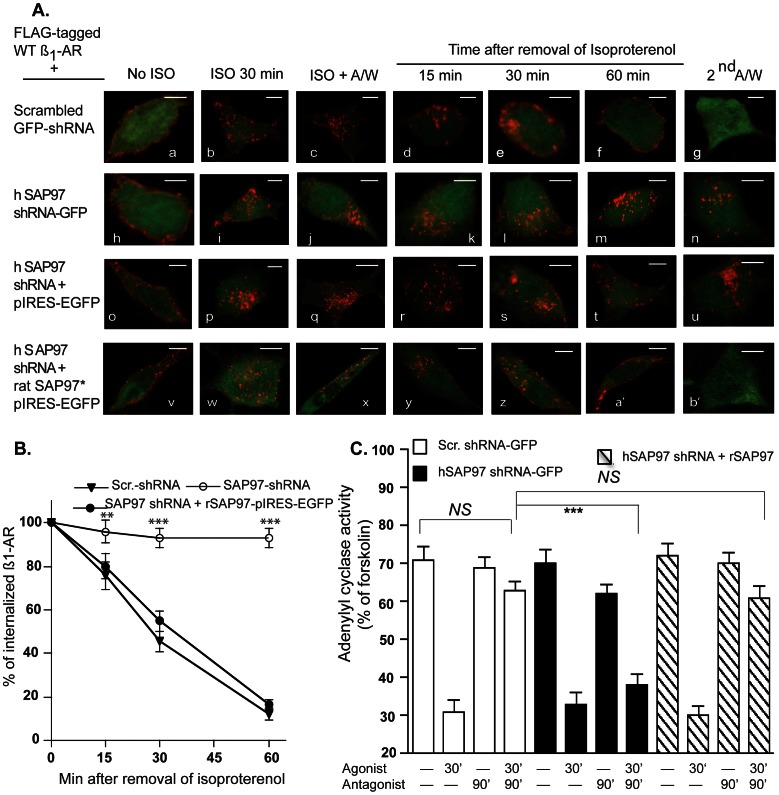
Effects of SAP97 knockdown and rescue on recycling and resensitization of the human ß_1_-AR. *A*, in *images a–n*, HEK-293 cells stably expressing the FLAG-tagged WT ß_1_-AR and either the scrambled shRNA-EGFP (*a–g*), or hSAP97 shRNA-EGFP (*h–n*) were used. In *images o-b’*, HEK-293 cells stably expressing the FLAG-tagged WT ß_1_-AR and hSAP97 shRNA were transfected with the empty pIRES-EGFP vector (*o–u*) or rSAP97 in pIRES-EGFPII (*v-b’*). Rat SAP97 constructs (denoted by an asterisk) are resistant to hSAP97 shRNA because of sequence mismatches in the region targeted by the human SAP97 shRNA. Cells on glass slides were prelabeled for 1 h with Cy3-anti-FLAG antibody and fixed (*images a, h, o, v*). The rest of the slides were exposed to 10 µM isoproterenol for 30 min (*b, i, p, w*) then acid washed and fixed (*c, j, q, x*). The rest of the slides were subjected to recycling conditions for the indicated time period and then fixed. Slides incubated with alprenolol for 1 h were exposed to acid wash and then fixed (*g, n, u, b’*). The distribution of fluorescent pixels was obtained using confocal microscopy and the colors shown are pseudo colors. *B*, pixels inside a 300-nm boundary in isoproterenol/acid-washed cells (*c, j, q and x*) were set arbitrarily to 100% to indicate 100% internalization and the ratios in alprenolol-treated cells were calculated and expressed as % for each time period. The ratios from 20 independent images for each condition were calculated and expressed as mean ± S.E. for each time period and then compared among the three different groups by one-way ANOVA with Newman-Keuls post-tests. Statistical results are expressed as (^*^), (^**^), and (^***^) *p*<0.05, *p*<0.01, and *p*<0.001, respectively. Each *scale bar* represents 5 µm. *C*, comparison of adenylyl cyclase activities in response to desensitization by isoproterenol and resensitization in HEK-293 cells. For the first two sets of experiments, cells stably expressing the WT ß_1_-AR with either the scrambled or the hSAP97 shRNA were used. For the third set, cells expressing the ß_1_-AR, SAP97 shRNA and rSAP97 in pIRES-EGFPII were used. Following the desensitization/resensitization protocol, cell membranes were prepared and used to measure adenylyl cyclase activities for each condition. These experiments were repeated *n = *3–5 times each in triplicate and were plotted as mean ± S.E. and then compared among different groups by one-way ANOVA with Newman-Keuls post-tests and expressed as described above. *NS*, indicates non significant difference among the compared groups.

To test whether the binding between SAP97 and the ß_1_-AR was a priori for recycling of the agonist-internalized receptor, we measured the effect of SAP97 knockdown and its rescue on trafficking of the WT ß_1_-AR. Scrambled or SAP97 shRNAs were created in the pcDNA 6.2 mir vector either as EGFP- tagged shRNAs or as untagged shRNAs. These constructs were expressed in HEK-293 cells that stably expressed the WT ß_1_-AR. In these double-stable cells, endogenous SAP97 was detected in cells expressing the scrambled shRNAs, but was selectively reduced by >80% in cells expressing the SAP97 shRNAs (data not shown). Next, we determined the effect of knock down of SAP97 on trafficking of the WT ß_1_-AR (Fig. 2A, *images a–n*). Cells stably expressing the WT ß_1_-AR with the scrambled EGFP-shRNA (Fig. 2A, *images a–g*) or with the EGFP-SAP97 shRNA (Fig. 2A, *images h–n*) were incubated with Cy3-labeled anti-FLAG M2-IgG (red, pseudo color) for 1 h and then visualized by confocal microscopy. Expression of either EGFP-shRNA did not interfere with the membranous distribution of the ß_1_-AR (Fig 2A, *images a and h*). Exposing these cells to isoproterenol resulted in rapid endocytosis of membranous ß_1_-AR into intracellular vesicles (Fig. 2A *images b* and *i*). After the removal of isoproterenol, the cells were incubated in an acetic acid/NaCl solution that stripped the surface-exposed antibody (ISO+A/W). This procedure revealed that internal ß_1_-AR were distributed into discrete punctate vesicular structures (Fig. 2A, *images c* and *j*). The effect of each shRNA on recycling of internal ß_1_-AR was initiated by removing isoproterenol followed by the addition of the ß-adrenergic receptor antagonist alprenolol to block further internalization, so that the recycling of internal ß_1_-AR could be unambiguously determined. In cells expressing the scrambled EGFP-shRNA, the agonist-internalized ß_1_-AR recycled back to the cell membrane within 60 min from the removal of isoproterenol (Fig. 2A *images d–f*). However, the ß_1_-AR did not recycle when SAP97 was knocked down (Fig. 2A *images k–m*). In cells expressing the scrambled EGFP-shRNA the ß_1_-AR recycled with a t_0.5_ = 18±5 min (Fig. 2B). The rate of recycling of the ß_1_-AR in these cells was comparable to the kinetics of ß_1_-AR recycling in native HEK-293 cells, indicating that the scrambled shRNA had no appreciable effect on recycling kinetics [9].

A major consequence of GPCR recycling is externalization, which involves the re-insertion of the internalized GPCR into the cell membrane [20]–[22]. If the externalized ß_1_-AR were inserted properly into the cell membrane, then Cy3-conjugated anti-FLAG IgG bound to the N-terminal FLAG epitope of the ß_1_-AR would be oriented extracellularly. In this case, a second acid wash would strip Cy3-anti-FLAG IgG from the externalized ß_1_-AR population [21]. Cells in which the ß_1_-AR was internalized and was then allowed to recycle for 60 min were exposed to a second acid wash (Fig. 2A *images g* and *n*). Acid wash of cells expressing the scrambled EGFP-shRNA stripped >80% of the Cy3 pixels indicating that recycled ß_1_-AR were externalized (Fig. 2A *image g*). On the other hand, Cy3 pixel distribution in SAP97 knockdown cells was not altered by this maneuver, indicating that the ß_1_-AR remained inside the cells that expressed the hSAP97 EGFP-shRNA (Fig. 2A compare *image g* to *n*).

To determine if rat SAP97 could rescue the recycling of the WT ß_1_-AR in SAP97 knock down cells, we used HEK cells stably expressing FLAG-tagged ß_1_-AR and untagged hSAP97 shRNA (Fig. 2A *images o-b’*). Rat SAP97 is resistant to hSAP97 shRNA because of sequence mismatches in the shRNA-targeted region. These cells were transiently transfected with either the empty p-IRES-EGFPII vector (Fig. 2A *images o-u*) or with rat SAP97 in pIRES-EGFP (Fig. 2A *images v-b’*). pIRES-EGFP is a bicistronic vector that allows the expression of the gene of interest to be monitored at the single-cell level due to expression of hrGFP II (green) on the same transcript. Images of cells that were pre-incubated with Cy3-labeled anti-Flag M2 IgG (red) for 1 h revealed that expression of empty pIRES-EGFP or rSAP97 in pIRES-EGFP did not interfere with the membranous distribution of the ß_1_-AR (Fig 2A *images o, v*). Exposing these cells to isoproterenol resulted in rapid endocytosis of membranous ß_1_-AR into intracellular vesicles (Fig. 2A *images p, w*). Expression of the empty pIRES vector did not rescue the recycling of the ß_1_-AR in SAP97 knock-down cells (Fig. 2A *images r–u*), while expression of rat SAP97 rescued the recycling of the ß_1_-AR and promoted its externalization (Fig. 2A *images y-b’*). Expression of rSAP97 in SAP97 knockdown cells rescued the recycling of the ß_1_-AR, which recycled with a t_0.5_ of 21±6 min (Fig. 2B). Analysis of the recycling data of Fig. 2B by one-way ANOVA indicated that the recycling kinetics of the ß_1_-AR in scrambled shRNA expressing cells *vs.* its recycling in SAP97 shRNA/rSAP97 expressing cells were comparable (*p*>0.05). On the other hand the recycling kinetics of the ß_1_-AR in these two cell types *vs.* the cell line expressing the SAP97 shRNA were significantly different (*p*<0.01 or *p*<0.001), as determined one-way ANOVA with Newman-Keuls post-hoc tests.

The relationship between ß_1_-AR externalization and resensitization was examined by means of desensitization/resensitization assays of adenylyl cyclase activity in membranes prepared from the cells described in Fig. 2A. The effect of SAP97 knockdown on resensitization was assessed in HEK-293 cells stably expressing the WT ß_1_-AR and the scrambled or the SAP97 shRNA as described in the Materials and Methods section (Fig. 2C). To determine if the shRNAs affected desensitization, the activity of adenylyl cyclase was determined in membranes prepared from cells that were exposed to isoproterenol for 30 min. The data are presented as percent of maximal activation of adenylyl cyclase activity in these membranes by 5 µM forskolin, which amounted to 110±18 pmol/mg protein/min. There was significant desensitization of the ß_1_-AR signaling pathway in cells expressing the scrambled shRNA or SAP97 shRNA, indicating that these shRNAs did not affect this parameter. The resensitization assay involved the desensitization of the ß_1_-AR with isoproterenol followed by incubating the cells for 90 min with 100 µM of the ß-antagonist alprenolol to induce externalization of the ß_1_-AR and subsequent resensitization of its adenylyl cyclase activity [10], [23]. The resensitization of adenylyl cyclase was restored to near control levels in cells stably expressing the WT ß_1_-AR and scrambled shRNA (Fig. 2C). On the other hand, in cells expressing the WT ß_1_-AR and hSAP97 shRNA, functional resensitization of adenylyl cyclase activity was significantly lower than in the cells expressing comparable levels of the scrambled shRNA (*p*<0.001). The effect of rescuing SAP97 expression on resensitization of adenylyl cyclase activity was determined by transient overexpression of rSAP97 in pIRES-EGFPII in a double stable cell line that expressed the FLAG-WT ß_1_-AR, and hSAP97 shRNA (Fig. 2C). Expression of rSAP97 did not affect the desensitization of the ß_1_-AR in this cell line. However, expression of rSAP97 rescued ß_1_-AR-mediated activation of adenylyl cyclase by ∼89±8% and these levels were not significantly different from the activity of adenylyl cyclase in membranes prepared from resensitized cells expressing the scrambled SAP97 shRNA (*NS*, from *n* = 4 experiments each in triplicate).

### Characterization of the PDZ Domain of SAP97 that is Involved in Recycling of the Human ß_1_-AR

**Figure 3:**
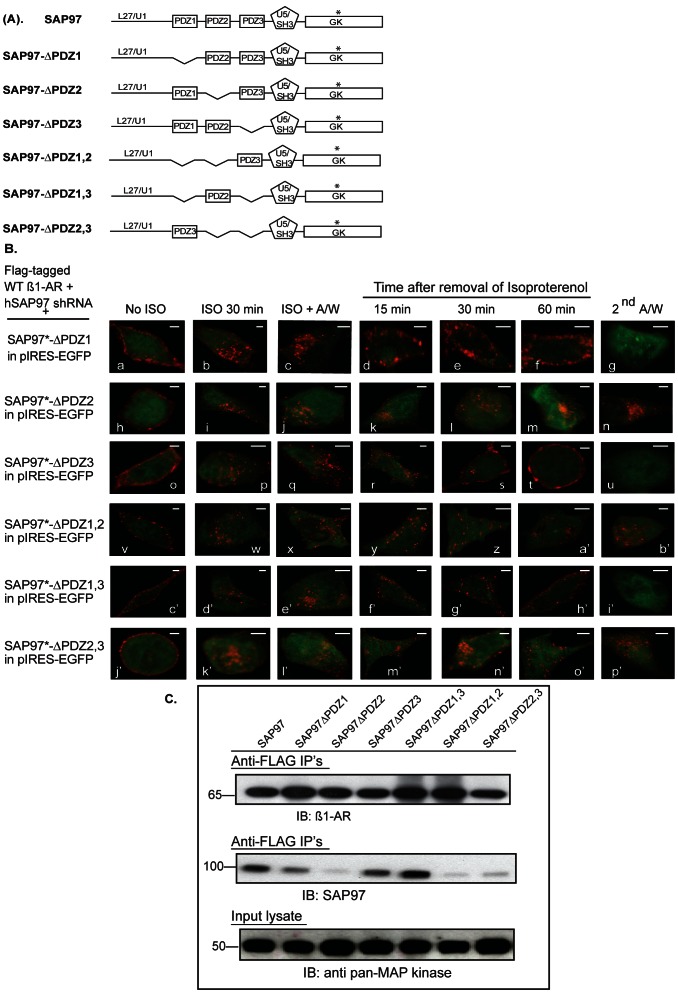
Mapping of the role of SAP97 PDZ domains in binding to and in recycling of the ß_1_-AR. *A*, schematic diagram of SAP97 and deletion constructs used in *panels B* and *C*. *B*, cells stably expressing FLAG WT ß_1_-AR and hSAP97 shRNA were transiently transfected with rSAP97 or its deletion constructs in pIRES-EGFP II that are shown in *panel 3A*. The internalization and recycling assays were carried out as described in the legend of Fig. 2. Each *scale bar* represents 5 µm. *C*, HEK-293 cells expressing hSAP97 shRNA and FLAG-tagged ß_1_-AR were transfected with pIRES-EGFP harboring SAP97 or its various PDZ deletion mutants shown in *panel A* above. Equal amounts of cell lysates (input) were incubated with ∼8 µL of anti-FLAG M2 IgG resin overnight. Then the beads were washed and eluted into 50 µl of 2X-Laemmli sample buffer. The top panel shows Western blot analysis of 10% of each FLAG IP that were probed with anti-human ß_1_-AR IgG (IB) to index ß_1_-AR IP’s. The middle panel shows Western blot analysis of 80% of each FLAG IP that were probed with anti-SAP97 IgG (IB) to index SAP97 Co-IPs. To normalize for input protein levels, ∼4% of each cell lysate were subjected to Western blotting (IB) and probed with the anti-pan MAP kinase antibody.

The effects of specific PDZ deletions in the full-length ß-isoform of SAP97 were generated in the pIRES-EGFP II vector (Fig. 3A). HEK-293 cells stably expressing the FLAG-tagged WT ß_1_-AR and hSAP97 shRNA were used in these studies. Each SAP97 deletion construct in pIRES-EGFP was transiently transfected into the double stable cell line and the trafficking of the ß_1_-AR was measured in EGFP expressing cells. In rSAP97 constructs with a single PDZ deletion such as ΔPDZ1 or ΔPDZ3, we observed normal recycling of internal ß_1_-AR and these receptors were externalized into the cells membrane (Fig. 3B *images d–g* and *r–u*). In cells expressing rSAP97 in which PDZ2 was deleted, internal ß_1_-AR failed to recycle and were retained intracellularly (Fig. 3B *images k–n*). In cells expressing double PDZ deletions, we observed that the ß_1_-AR did not recycle when PDZ2 was deleted such as in ΔPDZ1,2 or ΔPDZ2,3 (Fig. 3B *images y-b’* and *m’–p’*, respectively). However when PDZ2 was retained, such as in ΔPDZ1,3, the ß_1_-AR recycled normally and was externalized (Fig. 3B, *images f’ to i’*). These data indicate that PDZ2 was required for mediating the effect of SAP97 on recycling and reinsertion of the ß_1_-AR into the cell membrane.

Next we determined the interaction between the SAP97 constructs of Fig. 3A and the ß_1_-AR in HEK-293 cells. Cells doubly stable for the expression of FLAG ß_1_-AR and SAP97 shRNA were transiently transfected with plasmids expressing either full-length rSAP97 in pIRES-EGFP or the rSAP97 mutants described in Fig. 3A. Equal amounts of input cell lysates were incubated with anti-FLAG IgG resin overnight, followed by washing and eluting the resin. (Fig. 3C, lower *panel*) Resin eluates were divided into two portions, where by, ∼10% of each of the eluates were subjected to Western blotting and probed with the anti-ß_1_-AR antibody (Fig. 3C, *top panel*). These data indicated that equal amounts of FLAG-tagged ß_1_-AR were immunoprecipitated from each cell line. The remainder of each eluate (∼80%) were subjected to Western blotting and probed with anti-SAP97 to index SAP97 co-IP (Fig. 3C, *middle panel*). The data indicated that the ß_1_-AR co-IP’d rSAP97 from rSAP97 constructs that contained PDZ2, such as SAP97ΔPDZ1, ΔPDZ3 and ΔPDZ1,3. However, SAP97 constructs in which PDZ2 was deleted, such as ΔPDZ2, ΔPDZ1,2 and ΔPDZ2,3 were not co-IP’d by the ß_1_-AR. Therefore, the recycling data and the co-IP results indicated that PDZ2 was required for the interaction between SAP97 and the ß_1_-AR, which was a priori for recycling of the ß_1_-AR to occur.

### Characterization of the Involvement of other SAP97 Domains in Recycling of the Human ß_1_-AR

**Figure 4:**
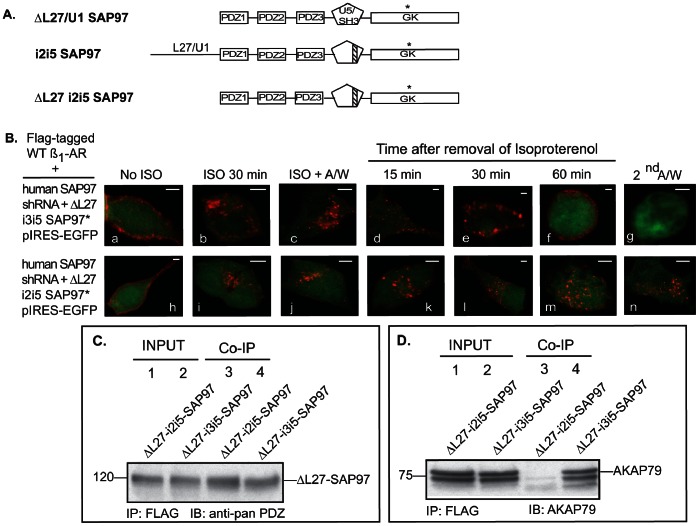
SAP97-AKAP79 interactions are involved in recycling of the ß_1_-AR. *A*, schematic diagram of SAP97 L27/U1 and U5 deletion constructs used in *panels B and C*. *B*, recycling of the ß_1_-AR in HEK-293 cells stably expressing hSAP97 shRNA was rescued by ΔL27-I3I5SAP97 but not by ΔL27 I2I5 ß-isoform of SAP97. Each *scale bar* represents 5 µm. *C–D*, ΔL27-I3I5 SAP97 or ΔL27-I2I5 SAP97 in pIRES-EGFP were transfected into the FLAG-tagged ß_1_-AR and hSAP97 shRNA double-stable cell line. In *C*, FLAG immunoprecipitations were probed with an anti-pan PDZ antibody to detect SAP97. In *D*, FLAG immunoprecipitations were probed with anti-AKAP79 antibody (IB). Input represented ∼4% of total cell lysate from each condition.

The ß-isoform of SAP97 contains at its N-terminus ∼200 amino acids L27 and a U1 domains that are involved in homo- and heterodimerization [26]. Cells expressing the hSAP97 shRNA were transfected with full length rat-SAP97 in pIRES-EGFP or a L27/U1 deletion mutant (ΔL27/U1-rSAP97 in pIRES-EGFP) and then examined for ß-agonist-mediated internalization and recycling of the ß_1_-AR (Fig. 4 A and B). In ΔL27/U1-rSAP97 expressing cells, the ß_1_-AR was internalized in response to isoproterenol and the agonist-internalized ß_1_-AR recycled back and was externalized into the membrane with kinetics similar to that of full-length SAP97 (Fig. 4B *images d–g*).

SAP97 also interacts with AKAP79/150, which is an A-kinase anchoring protein that binds PKA, PKC and protein phosphatase 2B [18], [27]. We have previously determined that AKAP79/150 was involved in the recycling and resensitization of the ß_1_-AR [23]. Within SAP97 there is an alternatively spliced U5 domain, which lies between the SH3 and GK domains and contains peptide inserts, termed i2–i5 [18], [28]. Nikandrova *et al* [18] determined that AKAP79/150 specifically interacted with SAP97 containing I3I5 inserts, while SAP97 lacking the I3 domain such as SAP97-I2I5 did not bind AKAP79. Expression of ΔL27-rSAP97-I2I5 in SAP97 knockdown cells did not interfere with membranous distribution of the ß_1_-AR (Fig. 4B *image h*) or with its internalization by isoproterenol (Fig. 4B *images i–j*). However, internal ß_1_-AR did not recycle in cells expressing ΔL27-rSAP97-I2I5 and were retained by >85% intracellularly (Fig. 4B *images k-n*). On the other hand, internal ß_1_-AR recycled efficiently in cells expressing ΔL27-rSAP97-I3I5 (Fig. 4B *images d–g*).

To determine the binding profile of ΔL27 and ΔI3 mutants of SAP97 to the ß_1_-AR, we transfected the following constructs into the FLAG-tagged WT ß_1_-AR and hSAP97 shRNA double-stable cell line. We used SAP97-ΔL27/U1 in pIRES or a double deletion construct in which L27 and I3 were deleted (SAP97-ΔL27-I2I5 in pIRES-EGFP (Fig. 4C). It was necessary to use a ΔL27 deletion construct in conjunction with the ΔI3 construct because expression of full-length SAP97-I2I5 was low and deletion of L27 increased its expression (unpublished observations). FLAG ß_1_-AR IP’s were probed for co-IP of these SAP97 mutants using an anti-pan PDZ antibody because the antigenic epitope of our SAP97 antibodies was in the L27 domain (Fig. 4C). IP’s of FLAG ß_1_-AR, co-IP’d both of these constructs, indicating that deletion of L27 or I3 did not affect the interaction between SAP97 and the ß_1_-AR.

Next, we tested the interaction of these constructs with AKAP79 (Fig. 4D). FLAG ß_1_-AR IP’s that were used in Fig. 4C were probed for co-IP of endogenous AKAP79 from these cells. FLAG IP’s from cells expressing ΔL27-SAP97-I3I5, co-IP’d AKAP79 (Fig. 4D, *lane* 4). However, FLAG IP’s from cells expressing ΔL27-SAP97-I2I5 did not co-IP AKAP79 (Fig. 4D, *lane* 3). These data reiterate the findings of Gardner *et al* [10] that the binding between AKAP79 and the ß_1_-AR was indirectly mediated by SAP97. Thus, deleting the I3 domain from SAP97 interfered with its binding to AKAP79 without affecting its binding to the ß_1_-AR. The data in figures 4C and 4D when combined with the recycling data of Fig. 4B demonstrate that binding of AKAP79/150 to SAP97 was also required for the recycling of the ß_1_-AR.

### Characterization of the Interaction between PDZ2 of SAP97 and the ß_1_-AR

**Figure 5:**
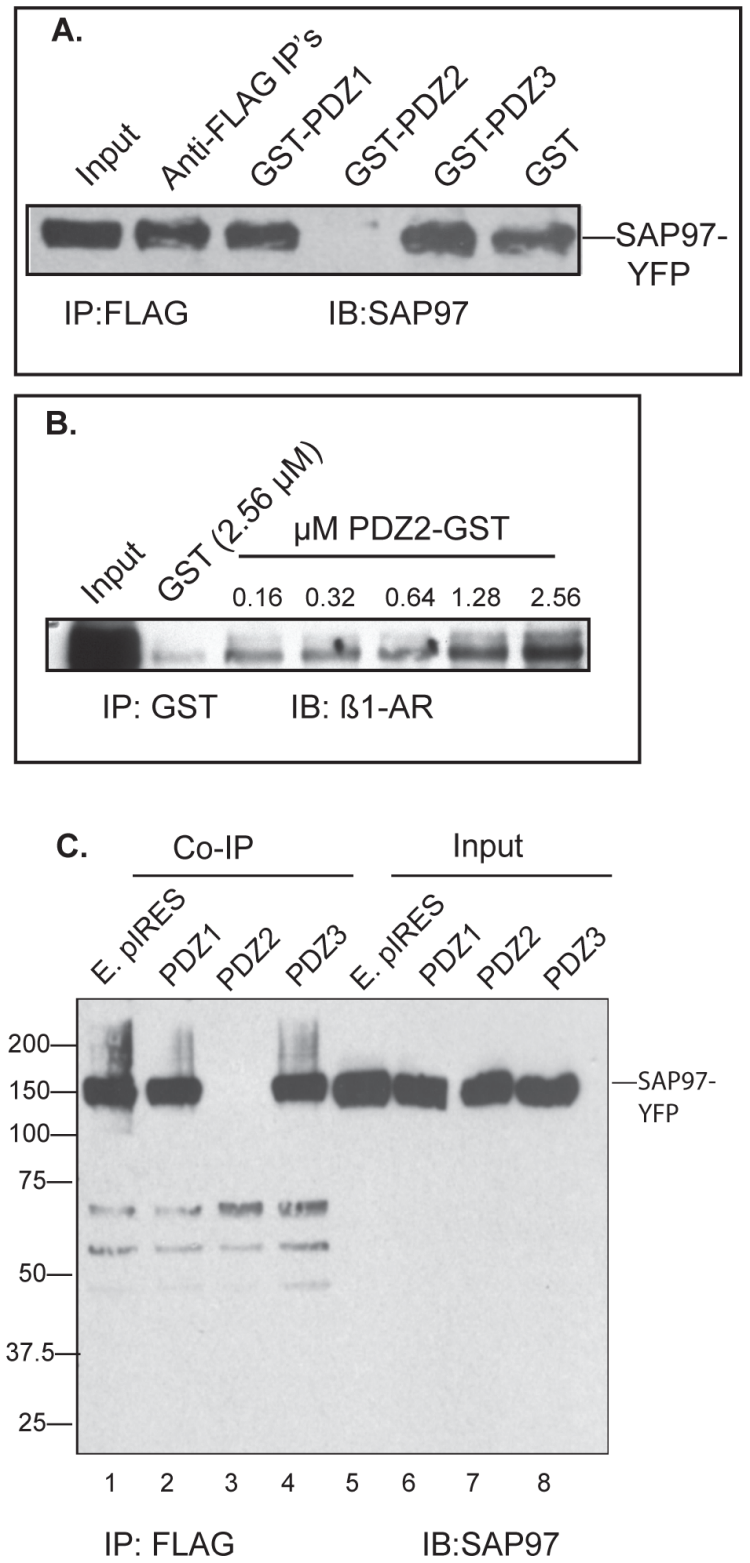
PDZ2 interference with the binding between SAP97 and the ß_1_-AR. *A*, in vitro interference of PDZ2 in the binding between SAP97 and the ß_1_-AR. Equal amounts of extract prepared from cells co-expressing FLAG ß_1_-AR and SAP97-YFP was mixed with 2.5 µg purified GST or 3.2 µg of GST-fusions of individual SAP97 PDZs in a total volume of 1 ml (0.1 µM). FLAG IP’s were subjected to Western blotting (IB) and probed with anti-SAP97. *B*, determining the affinity of the interaction between PDZ2 and the ß_1_-AR. Membranes from HEK-293 cells expressing 1.2 pmoles of ß_1_-AR per mg protein were solubilized and mixed with the indicated concentrations of GST of GST-PDZ2. GST-pull downs were probed with the anti-ß_1_-AR antibody and optical densities were used to calculate the EC_50_ for their interaction. *C*, in vivo interference of PDZ2 with ß_1_-AR binding to SAP97. HEK-293 cells expressing the FLAG ß_1_-AR and YFP-SAP97 were transfected with the indicated SAP97 PDZ in pIRES-EGFP. FLAG IP’s or ∼4% of input lysate were subjected to Western blotting (IB) and probed with anti-SAP97 antibody (*n = *3).

Initially we determined by in vitro interference the effect of the individual PDZs of SAP97 on the binding between the ß_1_-AR and SAP97. Extracts prepared from cells stably expressing FLAG ß_1_-AR and SAP97-YFP were mixed with 2.5 µg of GST or molar equivalent of GST-PDZ (Fig. 5A). Then anti-M2 FLAG IgG resin was added to determine if SAP97 was co-IP’d with the ß_1_-AR. These experiments indicated that PDZ2 was the only PDZ to interfere with the binding between the ß_1_-AR and SAP97.

To determine the affinity of PDZ2-binding to the ß_1_-AR, we mixed increasing amounts of GST-PDZ2 with known amounts of solubilized ß_1_-AR prepared from cells stably expressing 1.2 pmoles of FLAG ß_1_-AR per mg protein (Fig. 5B). These experiments indicated that the estimated EC_50_ of the interaction between GST-PDZ2 and the ß_1_-AR was ∼1±0.23 µM (*n* = 3). The result of GST pull down in Fig. 5B was comparable with the data of the overlay assay in Fig. 1B, indicating that the binding between SAP97 and the ß_1_-AR was mediated by PDZ2. Finally, we determined if in vivo overexpression of PDZ2 would interfere in the binding between the ß_1_-AR and SAP97. Each individual PDZ of SAP97 in pIRES-EGFP was transfected into HEK cells stably expressing the FLAG ß_1_-AR and SAP97-YFP (Fig. 5C). FLAG IP’s prepared from cells expressing empty pIRES (E. pIRES), PDZ1 and PDZ3 co-IP’d SAP97, demonstrating that these PDZs did not interfere with in vivo interactions between the ß_1_-AR and SAP97 (Fig. 5B *lanes 1, 2* and *4*). However, FLAG IP’s from cells expressing PDZ2 did not co-IP SAP97, indicating that PDZ2 interfered in the binding between the ß_1_-AR and SAP97 (Fig. 5B, *lane* 3).

**Figure 6:**
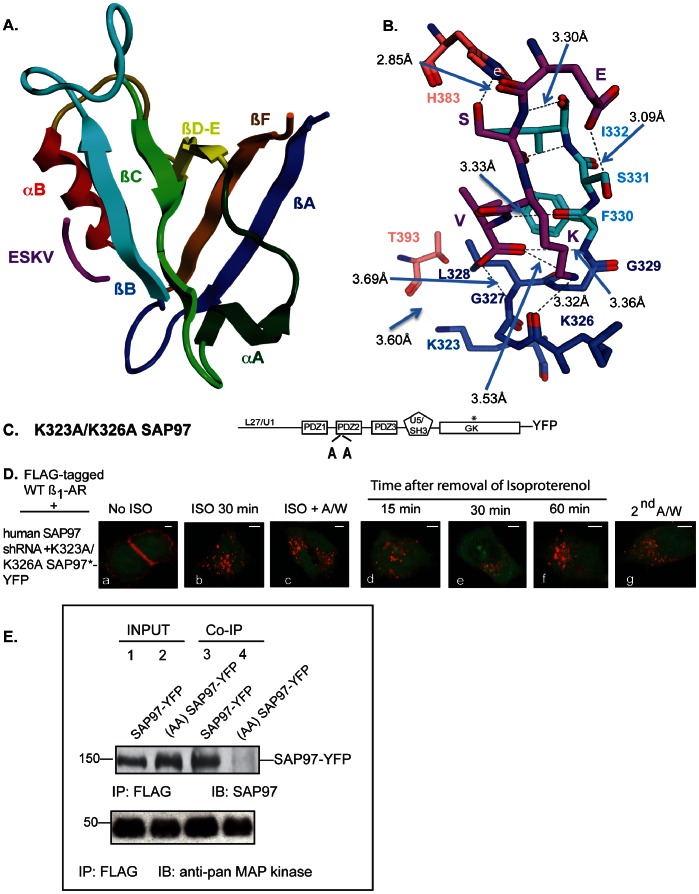
Lysines 323 and 326 in PDZ2 influence SAP97-mediated recycling and binding to the ß_1_-AR. *A*, ribbon diagram of the SAP97 PDZ2 structure with ESKV peptide bound. In PDZ2 domain helixes and strands are named according to Doyle *et al* [45] and shown in different colors. ESKV peptide is shown in magenta. *B*, ligand binding interactions between the C-terminal sequence (ESKV) of the human ß_1_-AR and SAP97 PDZ2 domain. Amino acids representing different strands and helixes in PDZ2 domain structure are shown in different colors: ßC strand, green; ßB strand, light blue; ßA strand, dark blue; alpha/B helix, light red. ESKV sequence is shown in magenta, oxygen atoms are depicted in red, nitrogen atoms are in navy blue and hydrogen bonds are indicated as dashed blue lines. Distance between the alpha-carbons of amino acids is shown in Å. *C*, cartoon of the K323A/K326A PDZ2 mutant that was used in *Panel D*. *D*, effect of mutating K323 and K326 to Ala on SAP97-mediated regulation of ß_1_-AR recycling. HEK293 cells stably expressing the FLAG-tagged WT ß_1_-AR and hSAP97 shRNA were transfected with K323A/K326A SAP97-YFP and the internalization and recycling of the ß_1_-AR were analyzed as described in Fig. 2. Each *scale bar* represents 5 µm. *E*, Cells stably expressing FLAG ß_1_-AR and hSAP97 shRNA were transfected with SAP97-YFP or K323A/K326A SAP97-YFP. FLAG IP’s from cell extracts were subjected to Western blotting (IB) and probed with anti-SAP97 antibody or with anti-pan MAP kinase antibody.

Physical interactions between PDZ domains and PDZ type-1 ligands mainly involve the binding of the terminal carboxylates of the hydrophobic residue at P = 0 in the PDZ type-1 ligand with the ßB domain of PDZ2, as well as binding between the serine/threonine at P = -2 with a highly conserved histidine in the alpha/B alpha-helix of PDZ2 (Fig. 6A and B) and [25], [29], [30]. These interactions are further stabilized by the binding of positively charged residues, such as the two positively charged lysines K323 and K326 in the binding pocket of PDZ2 (Fig. 6B). Mutagenesis of K323 and K326 to alanines inhibited the binding between PDZ2 and the GluR1 subunit of AMPA-type glutamate receptors [31]. To find out if these lysines were also involved in SAP97-mediated rescue of ß_1_-AR recycling, K323A/K326A rSAP97-YFP was expressed in SAP97 knockdown HEK cells (Fig. 6C and 6D). This construct did not interfere either with membranous distribution of the ß_1_-AR or with its internalization by isoproterenol (Fig. 6D *images a–c*). However, the K323A/K326A SAP97 construct could not rescue the recycling of internalized ß_1_-AR and these receptors were retained by >80% intracellularly (Fig. 6D *images d–g*).

Next we determined whether the K323A/K326A mutant would bind to the ß_1_-AR by transiently transfecting WT rSAP97-YFP or K323A/K326A rSAP97-YFP into cells stably expressing FLAG ß_1_-AR and hSAP97 shRNA. FLAG IP’s of cell extracts prepared from these cells, indicated that WT rSAP97-YFP was co-IP’d with the ß_1_-AR, but K323A/K326A rSAP97-YFP was not IP’d (Fig. 6E compare *lane* 3 to *lane* 4). These data show that lysines 323 and 326 of SAP97 PDZ2 were involved in SAP97 binding to the ß_1_-AR. To strengthen this argument we modeled the binding between the ESKV sequence corresponding to the last four amino acids in the carboxy tail of the ß_1_-AR to the published coordinates for the crystal structure of PDZ2 in complex with the C-terminal peptide (ATGL) of GluR1 [25]. The data in Fig. 6B indicated that the binding between SAP97 PDZ2 and the ESKV peptide involved the same amino acids that were involved in the binding between SAP97 PDZ2 and the ATGL peptide and that K326 was apparently involved in the binding.

### PDZ2 of SAP97 Inhibits the Recycling and Resensitization of the ß_1_-AR by Acting as a Dominant Negative Inhibitor of Endogenous SAP97

**Figure 7:**
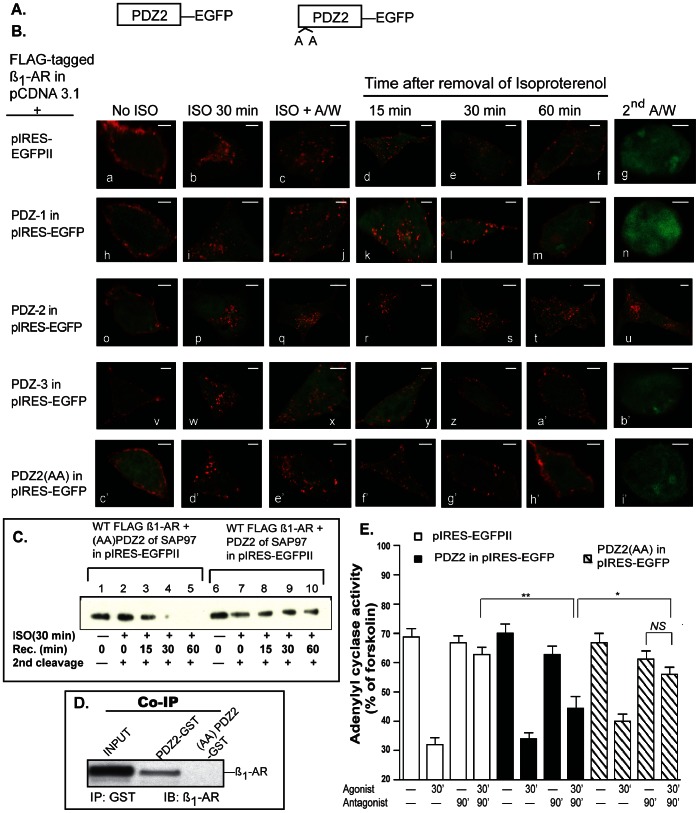
Effect of the isolated PDZs of SAP97 on trafficking and resensitization of the ß_1_-AR. *A, B*, each isolated PDZ of SAP97 in pIRES-EGFP was co-transfected into cells stably expressing the FLAG ß_1_-AR followed by determining their effect on internalization and recycling of the ß_1_-AR by confocal microscopy. Each *scale bar* represents 5 µm. *C,* Effect of overexpressing PDZ2 or K323A/K326A PDZ2 on recycling of the ß_1_-AR as determined by surface-biotinylation recycling assays. HEK-293 cells expressing the WT ß_1_-AR and K323A/K326A PDZ2 in pIRES-EGFP (*lanes 1–5*) or the WT ß_1_-AR with WT PDZ2 in pIRES-EGFP (*lanes 6–10*) were surface biotinylated and one set of plates was removed to determine total biotinylation for each cell line (*lanes 1* and *6*). The remaining sets of culture dishes were exposed to isoproterenol for 30 min at 37°C to induce ß_1_-AR internalization, followed by removal of isoproterenol and cleavage of the remaining surface biotin (1^st^ cleavage) with glutathione (*lanes 2–5* and *7–10*). One set of plates was extracted to determine the amount of internalized biotinylated ß_1_-AR (*lanes 2* and *7*). The remaining cultures were switched to warm culture medium supplemented with 10 µM ß-adrenergic receptor antagonist alprenolol and then returned to 37°C to allow the internalized ß_1_-AR to recycle for 15, 30, and 60 min. After each time period, the cells were recleaved (2^nd^ cleavage) to ensure cleavage of any newly appearing (recycled) surface biotin. After the second cleavage, the cells were solubilized in RIPA buffer, and equal amounts of clarified lysate protein were mixed with 50 µl of bovine serum albumin-blocked ultralink-neutra avidin beads at 4°C overnight. The beads were eluted and the eluates were probed by Western blotting using the anti-FLAG antibody. *D*, PDZ2-GST or PDZ2 (K323A/K326A)-GST were mixed with lysates prepared from cells expressing the wild-type ß_1_-AR, followed by analyzing the GST-pull downs for co-IP of the ß_1_-AR. *E*, effect of PDZ2 or K323A/K326A PDZ2 on desensitization and resensitization of ß_1_-AR-regualted adenylyl cyclase activity. Cells stably expressing the WT ß_1_-AR with either the PDZ2-EGFP or K323A/K326A PDZ2-GFP were subjected to desensitization and resensitization as described in the legend of Fig. 2. The activities of adenylyl cyclase in *n* = 5 experiments, each in triplicate were plotted as mean ± S.E. and then compared among different groups by one-way ANOVA with Newman-Keuls *post-hoc* tests. Statistical results are expressed as (^*^) and (^**^) to indicate *p*<0.05, *p*<0.01, respectively. *NS*, indicates non significant difference among the compared groups.

In this series of experiments we sought to determine the effect of each isolated PDZ of SAP97 on trafficking of the ß_1_-AR. We hypothesized that if PDZ2 binds selectively to the ß_1_-AR, then its overexpression would destabilize the ß_1_-AR-SAP97 scaffold and interfere with trafficking of the ß_1_-AR. Expression of the empty pIRES-EGFP vector or its fusions with individual PDZ’s did not affect the membranous distribution of the ß_1_-AR (Fig. *7B* *images a, h, o* and *v*) or its internalization in response to isoproterenol (Fig. 7B *images c, j, q* and *x*). Expression of isolated PDZ1 or PDZ3 did not interfere with the recycling of the WT ß_1_-AR or with its externalization (Fig. 7B, *images k–n* and *y-b’*). Expression of isolated PDZ2 however, prevented efficient recycling of internal ß_1_-AR (Fig. 7B *images r-t*). Acid treatment of these cells did not strip the Cy3 pixels, indicating that the majority (88±7%, *n* = 30) of internal ß_1_-AR remained inside the cell (Fig. 7B *image u*). These data show that PDZ2 interfered with the binding of the ß_1_-AR to SAP97 (Fig. 5B) and interfered with it’s recycling (Fig. 7B). Another method to assess the effect of PDZ2 on the internalization and recycling of the ß_1_-AR is to conduct these experiments on surface biotinylated HEK 293 cells expressing the WT ß_1_-AR and PDZ2-pIRES or K323A/K326A PDZ2-pIRES (Fig. 7C). An advantage of this method is its ability to assess the effect of PDZ2 on internalization and recycling of the ß_1_-AR in a larger population of cells. In these assays, cells were surface biotinylated with cleavable biotin followed by quenching of excess biotin with glycine. The amount of biotin incorporated into the ß_1_-AR under this condition indexed total cellular ß_1_-AR biotinylation (Fig. 7C *lanes 1* and *6*). The cells were then exposed to isoproterenol for 30 min, followed by cleavage of the remaining cell surface biotin (Fig. 7C *lanes 2* and *7*). The amount of biotin recovered in this step indexed the amount of biotinylated ß_1_-AR that was internalized in response to isoproterenol. Thus, the ratio of internal biotin to total biotin incorporated into the ß_1_-AR indexed the percentile of surface ß_1_-AR that was internalized in response to isoproterenol. When compared to WT PDZ2, it appeared that K323A/K326A PDZ2 reduced the amount of internalized ß_1_-AR in HEK-293 cells by ∼40% (compare *lane* 2 to *lane 7* in Fig. 7C). To determine the effect of PDZ2 on recycling of the agonist-internalized ß_1_-AR, isoproterenol was replaced with the ß-antagonist alprenolol to inhibit ß_1_-AR internalization (Fig. 7C *lanes* 3–5 and *lanes* 8–10). Internalized ß_1_-AR were allowed to recycle by warming the cells (at 37°C) for an additional 15, 30, or 60 min (Fig. 7C *lanes 3–5* and *8–10*). After each time period, the cells were cooled to 4°C and cleaved for the second time to cleave newly appearing surface biotin. Thus, the loss of biotin from the second cleavage step indexed recycling of the ß_1_-AR. The data indicate that by 30 min more than 90% of the biotin was lost from the ß_1_-AR in cells expressing K323A/K326A PDZ2 of SAP97, reflecting membrane recycling of the ß_1_-AR and subsequent biotin cleavage (Fig. 7C *lanes 3–5*). In contrast, internalized (biotinylated) ß_1_-AR in HEK cells expressing WT PDZ2 were not markedly changed even after 1 h from the removal of isoproterenol, reflecting their internal distribution (Fig. 7C compare *lanes 9* and *10* with *lanes 4* and *5*). The results of figures 7B and 7C show by two independent methods that an interaction between the ß_1_-AR and endogenous SAP97 via PDZ2 was required for recycling of the ß_1_-AR in HEK-293 cells.

Pull down assays between PDZ2-GST or K323A/326A-PDZ2-GST and the ß_1_-AR indicated that mutagenesis of K323 and K326 to alanine abrogated the ability of PDZ2 to pull down the ß_1_-AR (Fig. 7D). The effect of PDZ2 on recycling of the ß_1_-AR was mediated in part by the pair of lysines at positions 323 and 326 because co-expression of K323A/K326A-PDZ2 with FLAG ß_1_-AR did not markedly interfere with the recycling or externalization of the ß_1_-AR in HEK-293 cells (Fig. 7B *image h’* and Fig. 7C *lanes* 3–5).

To determine the effect of PDZ2 on resensitization of the ß_1_-AR, we transfected cells stably expressing the WT ß_1_-AR with empty pIRES-EGFP II, or with PDZ2 in pIRES-EGFP or K323A/K326A PDZ2 in pIRES-EGFP (Fig. 7E). Agonist-mediated desensitization of adenylyl cyclase activity was observed in all these cell lines. In the resensitization component of the assay and as expected, the activity of adenylyl cyclase in membranes prepared from cells expressing the empty pIRES vector was close to that in control untreated membranes (Fig 7E). Overexpression of PDZ2 markedly reduced the resensitization of the ß_1_-AR signaling pathway. The activity of resensitized adenylyl cyclase in membranes prepared from cells expressing the empty pIRES vector was 63±6% of maximal activity as indexed by forskolin. However, the comparable activity of adenylyl cyclase in membranes prepared from PDZ2 expressing cells was reduced to 44±7%, which was significantly lower than in control membranes (*n* = 4, one-way ANOVA, *p<*0.01). The activity of resensitized adenylyl cyclase in membranes prepared from cells expressing K323A/K326A PDZ2 was 56±6% of maximal activity, which was not significantly different from the 67±7% activity in membranes prepared from non-desensitized K323A/K326A PDZ2 expressing cells (*n* = 5). Comparisons between the activities of adenylyl cyclase in membranes prepared from PDZ2 resensitized cells versus their K323A/K326A PDZ2 counterpart indicated that the activity of adenylyl cyclase in K323A/K326A PDZ2 membranes was significantly higher (*n* = 5, *p*<0.05). Therefore, mutagenesis of K323A/K326A in PDZ2 interfered with the dominant negative activity of PDZ2 on recycling and resensitization of the ß_1_-AR signaling pathway.

**Figure 8:**
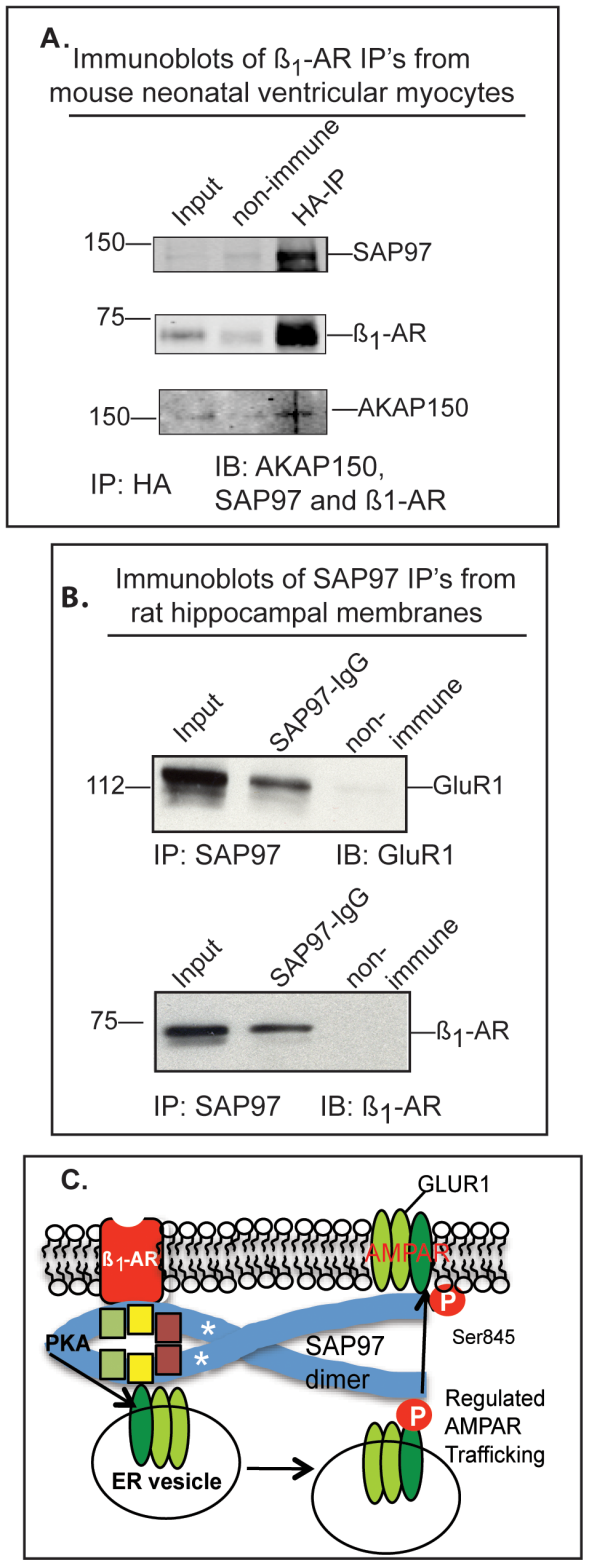
Characterization of molecular interactions between the ß_1_-AR and SAP97 and AKAP79/150 in cardiac and neuronal tissue. *A*, mouse neonatal cardiac myocytes were infected with an adenovirus harboring HA-tagged mouse ß_1_-AR. Then input lysates were immunoprecipitated with anti-HA resin (IP). The resin was eluted and equal amounts of eluate were subjected to Western blotting (IB) and probed with antibodies to AKAP150, SAP97 and ß_1_-AR. *B*, lysates of rat hippocampal membranes were incubated with 1∶200 dilution of anti-SAP97 IgG (Enzo Life sciences) or mouse IgG for 16 h at 4°C. Then 30 µl of protein G-agarose was added for 2 h, followed by 4 washes of the resin in complete RIPA buffer. The beads were suspended in 2X Laemmli sample buffer with 20 mM dithiothreitol for 45 min at 37°C. The cleared eluates were divided equally and subjected to SDS-PAGE on 8% (GluR1) or 10% (ß_1_-AR) gels followed by electroblotting to nitrocellulose membranes. These membranes were probed with anti-GluR1 (sc-28799) or ß_1_-AR (sc-568) antibodies from Santa Cruz Biotechnology. C, diagram representing the binding of SAP97 dimers to the ß_1_-AR in postsynaptic membranes and to the GluR1 subunit of AMPAR in a postsynaptic membrane specialization commonly categorized as the “perisynaptic membrane”. We hypothesize that activation of the ß_1_-AR signalosome ultimately activates PKA, which in turn phosphorylates the GluR1 subunit of AMPA receptors on Ser^845^ to initiate regulated trafficking of AMPA receptors from perisynaptic membranes to postsynaptic membranes [39]–[41].

The association between SAP97 and ß_1_-AR was explored in cells derived from neonatal mouse ventricular myocytes that endogenously express the ß_1_-AR, SAP97 and AKAP150, which is the rodent homolog of AKAP79 [32]. These cells were infected with adenovirus harboring the HA tagged ß_1_-AR then solubilized and the ß_1_-AR was immunoprecipitated from the clarified lysates with anti-HA agarose (Fig. 8A). Immunoprecipitation of HA-ß_1_-AR from these cells co-immunoprecipitated SAP97 and AKAP150 indicating that an association between the ß_1_-AR and SAP97 and AKAP150 occurred in mouse cardiac cells (Fig. 8A). Moreover, SAP97 IP’s from crude rat hippocampal membranes immunoprecipitated the GluR1 subunit of AMPA receptors as well as the ß_1_-AR (Fig. 8B). These data indicate that the ß_1_-AR was associated with SAP97 in both cardiac and neuronal tissues.

## Discussion

SAP97 is a major post-synaptic protein with broad distribution in the periphery [33]. SAP97 interacted with several receptors, channels and other scaffolding proteins to form and stabilize post junctional complexes [34]. SAP97 homodimerizes via its L27 domain to assemble these complexes into networks [16]. Previously we have shown that the type-1 PDZ ligand at the carboxy-tail of the ß_1_-AR was the binding site for SAP97 [10]. Moreover, inactivation of the ß_1_-AR PDZ abrogated SAP97 binding and inhibited the recycling and resensitization of the ß_1_-AR [9], [10]. In this report we demonstrated that knock down of SAP97 inhibited the recycling of internal ß_1_-AR as well as functional resensitization of the ß_1_-AR signaling pathway. On the other hand re-expression of siRNA-resistant rat SAP97 restored these functions, indicating that SAP97 was involved in mediating these effects.

SAP97 binding to the ß_1_-AR type-1 PDZ ligand promoted the formation of a ternary complex with AKAP79 that that we have termed the ß_1_-adrenergic receptosome [10]. Structurally, this model envisioned SAP97 to be sandwiched between the ß_1_-AR and AKAP79/150 (Fig. 8A) and [10]. The formation of this trimer was verified by co-immunoprecipitations from mouse neonatal cardiac myocytes (Fig. 8A) and by co-localization confocal microscopy in sympathetically innervated cardiac myocytes [32]. To better understand the contribution of each member of the ß_1_-adrenergic receptosome to its functions, we sought to determine the specific contributions of SAP97 to the overall organization of this complex and to its physiological functions. We concentrated first on identifying the PDZ-binding domain(s) in SAP97 that interacted with the ß_1_-AR PDZ. The methodical analysis identified the PDZ2 domain of SAP97 as the primary binding cassette to the PDZ ligand in the ß_1_-AR. This finding is significant because PDZ3 was previously reported as the PDZ that binds to the ß_1_-AR [13]. The identification of PDZ2 of SAP97 as a binding partner of the ß_1_-AR adds one more major signaling molecule to an already impressive retinue of PDZ2 binding partners, that include GluR1 and GluR2, E6 polypeptide of human papillomavirus (HPV), the type-1 somatostatin receptor, potassium voltage channels and others [25], [29], [30], [34]–[36]. Many of these interactions are physiologically important. For example, GluR1 binding to PDZ2 of SAP97 played an obligatory role in GluR1-dependent regulation of dendrite growth, while the interaction between the HPV18-E6 polypeptide and PDZ2 of SAP97 targeted SAP97 for degradation [31], [37]. The PDZ ligand sequences in the carboxy-termini of the aforementioned proteins are: “ESKV” in the ß_1_-AR, “ESDV” in the NR2B subunit, “ETQV” in the HPV-18-E6 and “ATGL” in the GluR1 subunit [25, 29 30, 36]. The 3D crystal structure of PDZ2 bound to the C-terminal polypeptides of GluR1 and HPV18-E6 polypeptide showed that despite the apparent difference in the sequences of their carboxy termini, the C-terminal hydrophobic amino acid at position (P = 0) and the highly conserved Ser/Thr amino acid at position (P = −2) of both sequences were bound to the same residues in PDZ2 of SAP97 [25], [29]. For example, the terminal main carboxyl group of these peptides was well anchored with the main chain nitrogens of the highly conserved GLGF motif in PDZ2, while the highly conserved S/T amino acid was bound to His^383^ in PDZ2 of SAP97. In Fig. 6A and B, we show that substituting the last 4 amino acids of the ß_1_-AR resulted in an energetically stable model where Val at P = 0 and Ser at P = −2 were bound to the expected residues of PDZ2. In addition, Lys^323^ and Lys^326^ were involved in stabilizing the binding pocket of PDZ2 as it transitions from the free to the liganded conformation [25], [29]. Mutagenesis of Lys^323^ and Lys^326^ to alanine in PDZ2 of SAP97 abrogated the binding of PDZ2 to GluR1 and the ß_1_-AR, indicating that this pair of lysines played a prominent role in PDZ2-mediated interactions (Fig. 6 and 7) and [31].

Our hypothesis concerning PDZ2 was that if WT PDZ2 was expressed in >85% of the cells, it would compete effectively with endogenous SAP97 in binding to the ß_1_-AR to allow a statistically significant effect on resensitization to be observed [15]. Transient overexpression of WT PDZ2 prevented the resensitization of the ß_1_-AR in a statistically significant manner (*p*<0.01) indicating that there was a relationship between PDZ2-mediated inhibition of the binding between SAP97 and the ß_1_-AR and its effect on resensitization. After this determination, we proceeded to determine the effect of mutated PDZ2 (K323A/K326A) on resensitization of the ß_1_-AR. Our expectation was that overexpression of mutated PDZ2 would not affect the resensitization because the mutated PDZ2 did not bind to the ß_1_-AR (Fig. 7D). Resensitization of the ß-AR signaling pathway was maintained when mutated PDZ2 was overexpressed, but we had to repeat this experiment five times, each in triplicate, before the values for resensitization were significantly higher (*p*<0.05 by one-way ANOVA) than those of WT PDZ2.

The binding between the ß_1_-AR and SAP97 however, was not sufficient for mediating the functional effects of SAP97 on trafficking and on resensitization of the ß_1_-AR signaling pathway. This can be gleaned from the data of figure 4, where SAP97-I2I5, which is missing the 33 amino acid I3 insert did bind to the PDZ ligand of the ß_1_-AR and did not promote the recycling and externalization of internal ß_1_-AR. These data support the idea that SAP97 acted in part as a bridging molecule between the ß_1_-AR and I3-binding partners. One such binding partner is AKAP79/150, which upon binding to the ß_1_-AR-SAP97 complex targets PKA, PKC, and/or PP2B to the ß_1_-AR microdomain [18], [27], [38]. It should be noted that HEK-293 cells endogenously express both the I2 and I3 variants of the ß-isoform of SAP97 as well as AKAP79 in sufficient amounts to support the recycling of exogenously expressed ß_1_-AR [18]. There are reports that indicate that the I3 domain was involved also in targeting SAP97 to synapses. Thus the I3 domain possesses other functional attributes that are important for neuronal functions of SAP97 [39].

Thus far we have been concerned with characterizing the significance of biochemical interactions between SAP97 and other members of the ß_1_-adrenergic receptosome, but the functional significance of these interactions has not been adequately discussed. We and other groups have identified several functional attributes to the ß_1_-AR/SAP97/AKAP79 scaffold. For example, this scaffold was involved in externalization and resensitization of the ß_1_-AR [9], [10], [23]. Another functional consequence for these interactions was the role of the ß_1_-AR PDZ domain in the carboxy tail in confining the ß_1_-AR to the plasma membrane of H9C2 cardiomyocyte-like cells [15]. In H9C2 cells, the integration of the ß_1_-AR (via its PDZ) in a complex with SAP97 and AKAP79/150 was responsible for confining the ß_1_-AR in the cell membrane as measured by single-particle tracking microscopy [15]. In this report we extend these observations to show the formation of a complex between ß_1_-AR-SAP97 and AKAP79/150 in mouse cardiac myocytes (Fig. 8A). We also provided a molecular basis for this complex by showing that the PDZ2 and I3 domains of SAP97 were bound to the ß_1_-AR and AKAP79, respectively. This is the first demonstration that MAGUK proteins utilize multiple interacting cassettes to regulate the activities of a GPCR. Given that many GPCRs contain a PDZ ligand at their carboxy-termini, this funding highlight a novel mechanism by which their cognate MAGUK proteins could regulate the physiological activity of their GPCR partners.

A surprising finding was the lack of involvement of the L27 dimerization domain in regulating biochemical interactions between SAP97 and the ß_1_-AR or the recycling and functional resensitization of the ß_1_-AR. This should not imply that L27 is not involved in more complex functions of the ß_1_-AR, such as its targeting to specific cellular compartments in complex cells of cardiac or neuronal tissues, or in organizing supramolecular assemblies between the ß_1_-AR signaling pathway and other signaling cascades, as discussed below.

Thus far we have shown that SAP97 binding to the ß_1_-AR PDZ (via PDZ2) and to AKAP79 (via I3) recruited PKA to the ß_1_-AR. This model is similar to the model for targeting PKA to AMPAR through the association of the GluR1 subunit of AMPAR with SAP97 (via PDZ2) and with AKAP79 through SAP97 [31], [35], [39]. Moreover activation of hippocampal ß_1_-AR caused the activation of a pool of PKA in close proximity to the SAP97-GluR1 scaffold in perisynaptic membranes to faithfully phosphorylate GluR1 on Ser^845^ [40], [41]. Phosphorylation of GluR1 subunits of AMPA receptors increased their trafficking from perisynaptic membranes to postsynaptic membranes to promote LTP and other behavioral responses [41], [42]. Thus, it is conceivable that ß_1_-AR and AMPAR could be brought into close proximity through their shared binding to PDZ2 of SAP97 homodimers that are formed by L27 domain oligomerization (Fig. 8C). Recently, a model for the association between PSD-95 and the ß_2_-AR via PDZ3 of PSD-95 was reported [43]. In this model AKAP79/150 was associated with the ß_2_-AR and the complex of PSD-95-ß_2_-AR-AKAP79/150 was linked to GluR1 via stargazin to form a macromolecular complex between the ß_2_-AR and AMPAR. PSD-95 binds avidly to the ß_1_-AR PDZ domain, but unlike SAP97 the interaction between the ß_1_-AR and PSD-95 inhibited the internalization and recycling of the ß_1_-AR [10], [44]. Therefore, tissue-specific molecular complexes between GPCR and other signal transducing complexes might diversify GPCR signaling to affect complex functions outside its canonical signaling paradigms.
